# Overcoming Barriers to Diabetes Technology in Youth with Type 1 Diabetes and Public Insurance: Cases and Call to Action

**DOI:** 10.1155/2022/9911736

**Published:** 2022-03-01

**Authors:** Ming Yeh Lee, Molly L. Tanenbaum, David M. Maahs, Priya Prahalad

**Affiliations:** Division of Pediatric Endocrinology, Stanford University School of Medicine, Standford, CA, USA

## Abstract

Advancements in diabetes technology such as continuous glucose monitoring (CGM), insulin pumps, and automated insulin delivery provide opportunities to improve glycemic control for youth with type 1 diabetes (T1D). However, diabetes technology use is lower in youth on public insurance, and this technology use gap is widening in the US. There is a significant need to develop effective interventions and policies to promote equitable care. The dual purpose of this case series is as follows: (1) describe success stories of the CGM Time in Range Program (CGM TIPs), which removed barriers for initiating CGM and provided asynchronous remote glucose monitoring for youth on public insurance, and (2) advocate for improving CGM coverage by public insurance. We describe a series of six youths with T1D and public insurance who obtained and sustained use of CGM with assistance from the program. Three youths had improved engagement with the care team while on CGM and the remote monitoring protocol, and three youths were able to leverage sustained CGM wear to obtain insurance coverage for automated insulin delivery systems. CGM TIPs helped these youths achieve lower hemoglobin A1c and improved time in range (TIR). Despite the successes, expansion of CGM TIPs is limited by stringent barriers for CGM approval and difficult postapproval patient workflows to receive shipments. These cases highlight the potential for combining diabetes technology and asynchronous remote monitoring to support continued use and provide education to improve glycemic control for youth with T1D on public insurance and the need to reduce barriers for obtaining CGM coverage by public insurance.

## 1. Introduction

Sustained use of diabetes technology, such as continuous glucose monitoring (CGM) and insulin pumps, improves glycemic outcomes in youth with type 1 diabetes (T1D), thereby reducing the risk for short-term and long-term complications [1–10]. The 2021 American Diabetes Association (ADA) standards of care expanded recommendation of CGM use to all people with diabetes on rapid-acting insulin [11], and the International Society of Pediatric and Adolescent Diabetes (ISPAD) 2018 guidelines encourage CGM use [8]. Additionally, social determinants of health (SDOH) are increasingly acknowledged as an important target to achieve equity in diabetes care [12]. In the United States, diabetes technology use is lower in youth of lower socioeconomic status (SES), and the gap of technology use between the highest and lowest SES groups has widened in the past decade [13–16]. The disparity may be, at least in part, due to restrictions in coverage for CGM by public insurance [17]. Our study focuses on public insurance coverage by California Children's Services (CCS), a Medicaid supplement for children in California with certain chronic health conditions, including diabetes. Youth with CCS need to demonstrate four or more self-monitored blood glucose (SMBG) checks daily for a month for CGM approval, a requirement that does not exist for privately insured youth. Insulin pump approval requires four SMBG checks daily or consistent CGM use, which is typically ≥70% wear based on our experience. These requirements present systemic barriers to technology adoption in youth on public insurance and contribute to the disparity. The purpose of this case series is as follows: (1) describe success stories of the CGM Time in Range Program (CGM TIPs), which removed barriers for initiating diabetes technology and provided asynchronous remote glucose monitoring for youth on public insurance, and (2) advocate for improving CGM coverage by public insurance to improve diabetes care and as a gateway to automated insulin delivery (AID). Specifically, this case series highlights an urgent need to remove systemic barriers causing disparity to access diabetes technology, while larger scale studies on this topic are ongoing.

## 2. Methods

The six youths in this case series were identified by pediatric endocrinologists and clinical diabetes care and education specialists (CDCES) as having CCS and being unable to meet CCS criteria for CGM coverage or having inconsistent CGM coverage. The youths were enrolled in an IRB-approved clinical research program called CGM TIPs, which supports youth with T1D and public insurance to initiate and sustain CGM use. Philanthropic funding for this program allowed us to provide CGM supplies for initial weeks, while clinic staff assisted families to obtain insurance coverage for further supplies. For interested families who have met the CCS requirement to qualify for an insulin pump, which includes CGM wear for at least one month, the program also assisted families in obtaining insurance coverage for insulin pumps and AID systems. CDCES remotely reviewed youths' CGM data monthly and provided diabetes education and dose adjustments as needed according to previously published protocol from the 4T study [18]. Hemoglobin A1c (HbA1c) was also monitored quarterly per routine care.

## 3. Case

Youth 1 is a 14-year-old male with a four-year history of T1D on SMBG and multiple daily injection (MDI) insulin. His HbA1c was 12.1% prior to CGM TIPs. Barriers to diabetes management included that his family is primarily Spanish-speaking, had limited understanding of diabetes, and were not comfortable using smartphones. He did not like checking fingerstick glucoses, and the team was concerned that he was bolusing insulin without checking his blood glucose. In addition, his mother aimed to keep bedtime glucose above target range (>200 mg/dL) due to her worry about overnight hypoglycemia.

Outcome: with CGM TIPs, he obtained and sustained on CGM. He preferred using the sensor compared to SMBG and began taking insulin more consistently. The CGM data provided reassurance for his mother's fear of hypoglycemia, leading to increased time in target range. Seven months into CGM TIPs, his HbA1c improved to 7.1% with CGM TIR 65.

Youth 2 is a 20-year-old female with T1D for five years and Hashimoto thyroiditis. She did not like SMBG checks and struggled with consistent insulin injections. Her HbA1c was in the range of 9.3–14% prior to CGM TIPs. She lives with her father, who has not been involved with her diabetes care. As a high school student, she benefitted from school nurse support to ensure consistent insulin injections. Since graduating from high school, she no longer has a school nurse as a resource. She experiences difficulty obtaining diabetes supplies consistently from the pharmacy and reports challenges with bolusing consistently. She is successful at consistent delivery of long-acting insulin.

Outcome: since enrollment in CGM TIPs, she is more engaged in her diabetes care and discusses CGM data and diabetes management frequently with CDCES through the remote monitoring program. Six months into CGM TIPs, her HbA1c improved to 7.1% with CGM TIR 53%. As she transitions off CCS insurance after 21 years of age, continuing CGM coverage will likely be challenging.

Youth 3 is an 18-year-old female with T1D for four years with HbA1c persistently >14% prior to CGM TIPs. The youth's mother had a history of substance abuse and homelessness; she died soon after the youth's T1D diagnosis. This youth's family is primarily Spanish-speaking, so she manages calls with insurance and medical equipment suppliers. Since graduating high school, she no longer has access to school nurse support to promote consistent SMBG and boluses.

Outcome: nine months since enrolling in CGMTIPs, her HbA1c improved steadily to 6.3% with CGM TIR 82%. She was briefly off CGM due to device malfunction and running out of supplies early. The program bridged the gap with supplies until she received new supplies.

Youth 4 is a 17-year-old female with T1D for nine years and a history of disordered eating. For the past several years, she has lacked support and supervision at home for diabetes management. She had sole responsibility for SMBG, dosing insulin and obtaining diabetes supplies. She was on CGM with an AID system intermittently prior to CGM TIPs, but had several lapses in her supplies due to difficulty navigating the healthcare system to obtain refills. Her HbA1c ranged 12–13% when she was off CGM and reverted to open loop and improved to 9–10% on the AID system prior to CGM TIPs. Her CGM TIR varied widely from 19% to 65%.

Outcome: with coaching from CDCES through CGM TIPs, she gained healthcare navigation skills such as knowing when and how to contact device companies when devices malfunction, which enabled her to maintain access to supplies. She now consistently uses her AID system, and her latest HbA1c was 7.7% and CGM TIR was 62%.

Youth 5 is a 15-year-old male with a five-year history of T1D, as well as asthma, eosinophilic esophagitis, and celiac disease. Prior to CGM TIPs, his HbA1c was >14%, and he had nine intensive care admissions for diabetic ketoacidosis (DKA) in the past 2 years. Lack of stable housing and co-occurring anxiety and depression presented major challenges to this youth's diabetes management. Based on assessment from the clinical team, family barriers included limited health literacy and communication skills to navigate the healthcare system. SMBG, insulin doses, and clinic appointments occurred less frequently than recommended.

Outcome: through CGM TIPs, he initiated CGM and subsequently an AID system. He became more engaged in his diabetes care, including responding to high glucose alerts on his CGM with correction doses, bolusing for carbohydrates, and responding to CDCES messages to adjust insulin pump settings. His HbA1c improved to 11.5% with CGM TIR 32%. He has had no DKA admissions in the seven months since enrollment in CGM TIPs, despite having COVID-19. His family developed a strong therapeutic alliance with the care team and continues to have frequent contact with his primary CDCES and social worker.

Youth 6 is a 14-year-old male with T1D for five years and attention deficit hyperactivity disorder. He had three DKA admissions in the past year, during which he did not attend any clinic appointments. Prior to CGM TIPs, his HbA1c ranged 9.7–12%. Family was interested in CGM, but he was unable to meet CCS criteria of four SMBG checks daily. He was resistant to wearing CGM sensors and removed them due to discomfort.

Outcome: after CDCES worked closely with him to discuss benefits of CGM and optimize comfort, he has worn the sensor consistently for 10 months. He subsequently obtained an AID system through CCS. His HbA1c improved to 8.3%, with CGM TIR 39%, and he had no additional DKA admissions. Although CGM TIPs facilitated initiation of technology, the family continues to have difficulty navigating the healthcare system to sustain supplies, such as responding to supplier phone calls. The program has bridged the gaps by providing supplies and coaching the family on communication skills to maintain supplies.

## 4. Discussion

This case series demonstrates how six youths on public insurance with suboptimal management of T1D benefitted from CGM TIPs, a program that reduced barriers to CGM access and use, provided asynchronous remote glucose monitoring, and increased multidisciplinary team support for troubleshooting diabetes management. These cases identify how the program supported them to initiate and sustain diabetes technology with subsequent improvements in glycemic control. Based on our experience with CGM TIPs and in alignment with the most recent ADA Standards of Care [11] and ISPAD guidelines [8], we recommend changes in the US public insurance policies to cover CGM for youths with diabetes and without unrealistic barriers for initiation, similar to many other countries [10, 19–21].

CGM TIPs removed barriers to CGM initiation for youths with public insurance by providing CGM and CDCES support. We found associated improvement in engagement with care, reduction in HbA1c, sustained CGM use, and decrease in DKA admissions. In this case series, we observed reduction in fear of hypoglycemia, which is a previously described benefit of CGM technology [22]. The remote monitoring program provided frequent short touch-points for CDCES to help families troubleshoot diabetes management issues and learn to navigate the health system, such as calling suppliers monthly to reorder supplies, reaching out to suppliers for replacements, and contacting the diabetes team for medical issues. We also observed reduction in gaps in CGM supplies. Interruptions in CGM use in youth with public insurance is primarily due to gaps in insurance coverage and is associated with increased HbA1c [9]. CGM TIPs addressed this barrier by bridging gaps in supplies with philanthropic funding. All youths in this case series demonstrated reduction in HbA1c after enrollment in CGM TIPs, including impressive reductions from >14% to 7%. Two youths with prior frequent DKA admissions have had no admissions since CGM TIPs initiation.

SDOH are essential intervention targets to achieve equity in diabetes care [12]. Financial cost is a major obstacle to diabetes technology use [23]. In the US, youths of lower SES on public insurance are unlikely to afford CGM and insulin pumps without insurance coverage. They are also especially at risk for having low health literacy and technology literacy [24, 25]. Many of these youths also come from non-English speaking families or lack resources such as stable housing, transportation, and Internet connection. These factors contribute to challenges engaging with care and obtaining diabetes technology. Currently, CCS coverage for CGM requires demonstration of >4 SMBG checks daily for a month. This is an outdated policy given the most recent ADA [11] and ISPAD guidelines [8] and an unnecessary hurdle as CGM provides more data and safety without requiring finger sticks. The process of navigating the insurance system is especially challenging for non-English speaking families and youth without adequate family support. Currently, CGM TIPs has philanthropic support to supply CGMs for families until insurance coverage is approved and to bridge supplies during gaps in insurance coverage. Scalability of this program depends on removal of outdated criteria for CGM coverage by public insurance, reducing friction for obtaining supplies, and adequate support for youths and their families.

CGM use is a gateway to an AID system, a technology that can help youths achieve even better glycemic control with less burden of care [5, 26–28]. Real-world data evaluating youths on an AID system demonstrated decreased HbA1c over six months, with the greatest HbA1c decrease in participants with the highest baseline HbA1c [29]. Historically, many practices restricted access to diabetes technology to youths who were not checking SMBG regularly or who had elevated HbA1c due to concerns of risk of DKA. However, more recent data suggest lower rates of severe hypoglycemia and DKA with CGM and pumps [30, 31]. Unrestricted CGM coverage for youths with T1D on public insurance improves engagement and provides a gateway to obtaining AID systems to improve their diabetes management.

## Data Availability

The data used to support the findings of this study are available from the corresponding author upon request.
